# Isolated Bone Lesions in the Mandible and Maxilla of Langerhans Cell Histiocytosis Treated with Fractionated Stereotactic Low-Dose Radiotherapy: Case Report and 5-Year Follow-Up

**DOI:** 10.1155/2021/9972240

**Published:** 2021-08-03

**Authors:** Antoine Berberi, Georges Aoun, Georges Aad, Elie Azar

**Affiliations:** ^1^Department of Oral and Maxillofacial Surgery, Faculty of Dental Medicine, Lebanese University, Lebanon; ^2^Department of Oral Medicine and Maxillofacial Radiology, Faculty of Dental Medicine, Lebanese University, Lebanon; ^3^Department of ENT, Faculty of Medicine, Lebanese University, Lebanon

## Abstract

Langerhans cell histiocytosis is a benign histiocytic disorder touching both genders and can occur at any age. It is currently classified by the Histiocyte Society as an inflammatory myeloid neoplasm of mixed cellularity. Clinically, it is illustrated by single or multiple osteolytic bone lesions associated with ulceration of the skin and soft tissues. Disease outcome is highly variable, depending on the degree of involvement. Bone pathologies are observed in 60% of cases as uni- or multifocal lesions. Several treatment modalities have been proposed and include surgical excision, intralesion steroid injection, chemotherapy, and low-dose radiotherapy. In this paper, we report a case of a 42-year-old male suffering from gingival swelling in the left side of his mandible and the right side of the maxilla. Clinical, radiological, and histological examinations confirm the diagnosis of Langerhans cell histiocytosis. Hematological investigation, entire body CT scan, and bone scintigraphy confirmed the limitation of the lesions in the right maxilla and on the left mandible. The lesions were treated with fractionated stereotactic low-dose radiotherapy, 24 Gy in 16 fractions, by using a linear accelerator with a custom-made rigid mask for accurate immobilization of the head with confirmed precision, which allows noninvasive approaches. Complete remission was achieved clinically and radiologically after one year, and a panoramic X-ray after 5 years confirms the bone healing process. Fractionated stereotactic low-dose RT could be adopted as an effective treatment.

## 1. Introduction

Langerhans cell histiocytosis (LCH), named by the Histiocyte Society (HS) in 1987, previously identified as histiocytosis X (HX), is a hyperplastic cellular disease of undetermined causes [1]. LCH is categorized by an uncontrolled clonal proliferation of Langerhans cells, which is a part of the human mononuclear phagocytic system [2].

Usually characterized by single (monostotic) or multiple (polyostotic) osteolytic bone lesions, solitary or multiple lymph node involvements associated to ulcerations of skin and soft tissues, and involvement of the central nervous system (CNS) [3].

Bones, excluding hands and feet, are the most commonly affected organs (80%) with 75% unifocal lesions and painful soft tissue swelling [3–5]. LCH is divided into 3 types: acute, disseminated, chronic, multifocal, and chronic focal [6]. Subdivisions for localized and disseminated LCH are reported by the blood group in 2016 [2]. Treatment modalities for adults have never been clarified by a clinical trial and the clinical reports deliver minimal data on the efficiency of various adopted treatments, which include surgery/curettage, steroids, low-dose radiotherapy (RT), and various chemotherapy regimens [7–14].

In this manuscript, we reported a case of isolated osteolytic lesions related to HLC observed in both jaws and treated with fractionated stereotactic low-dose RT with a 5-year follow-up period.

## 2. Case Report

A 42-year-old male has been oriented to our department, complaining of unilateral facial swelling with persistent pain, one year after extraction of the left mandibular premolars.

Additionally, he presented a gingiva swelling in the right maxilla related to a fixed bridge.

**Figure 1:**
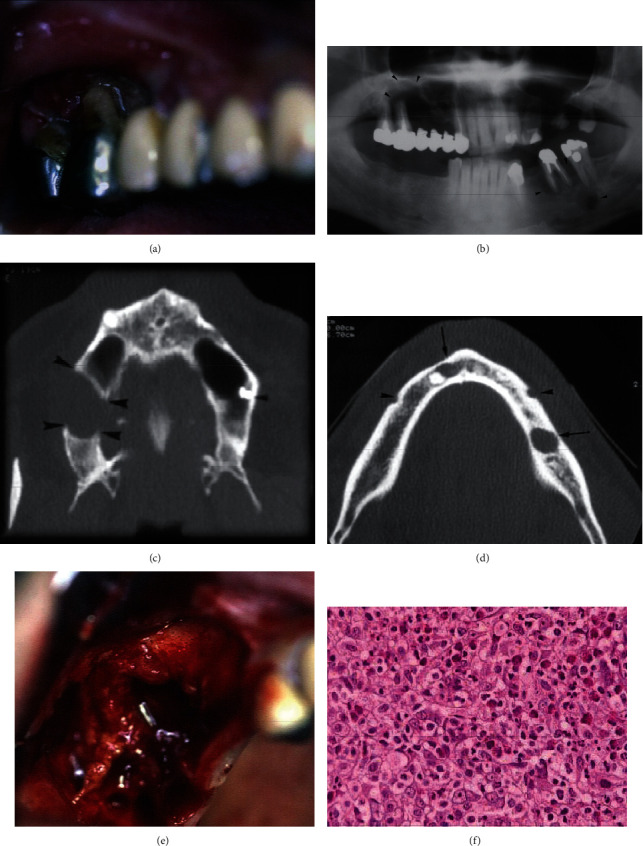
(a) Gingival ulcer proliferative growth in relation with the restored right maxillary molars. (b) 2D panoramic radiograph showing the unilocular radiolucency in the maxilla and the mandible. (1) Axial images of the CT scan exposed the presence of radiolucency in the maxilla with cortical bone deterioration. (d) axial images of the CT scan displaying a well-defined localized radiolucency in the body of the mandible. (e) The excised tissue after tooth extraction. (f) Histopathology of Langerhans cell histiocytosis: prominent histiocytes with abundant cytoplasm against the background of mixed population of eosinophils and multinucleated giant cells (HE ×10).

The clinical exam showed palpable lymph node, nontender, freely movable on the left submandibular area, and intraoral examination revealed a painful buccal cortical expansion in relation to the first and second mandibular molars. In the maxilla, a gingival ulcer proliferative growth in relation to the restored right molars was observed with a mobility type II (Figure 1(a)). A 2D panoramic radiograph was taken and showed a well-defined unilocular radiolucency in the apical area of the second maxillary molar with no extension to the maxillary sinus. In the left side of the mandible, two well-defined radiolucencies were noted, the first in relation to the mesial root of the first molar and the second on the mandibular body apically to the second molar (Figure 1(b)).

The axial images of the CT scan revealed the presence of radiolucency in the maxilla with cortical bone deterioration buccally and palatally and in the mandible and a well-defined localized radiolucency in the body of the mandible (Figures 1(c) and 1(d)).

Based on clinical and radiological findings, the differential diagnosis suggested was a radicular cyst, osteomyelitis, primary bone tumor, lymphoma, and LCH.

The extraction of the maxillary molars was proposed by us and approved by the patient. Under loco-regional analgesia, the two teeth were extracted, and the excised tissue was sent for histological evaluation (Figure 1(e)). The result came out with an LCH diagnosis (Figure 1(f)).

Histological examination exposed cellular proliferation characterized by unique reniform nuclei with eosinophilic cytoplasm representing Langerhans cells, in a stroma with numerous acute and chronic inflammatory infiltrate cells.

The patient was oriented to the internal medicine department to determine the degree of the disorder and to discuss the appropriate treatment protocol.

Hematological explorations displayed a significant increase in eosinophil count by 12%.

**Figure 2:**
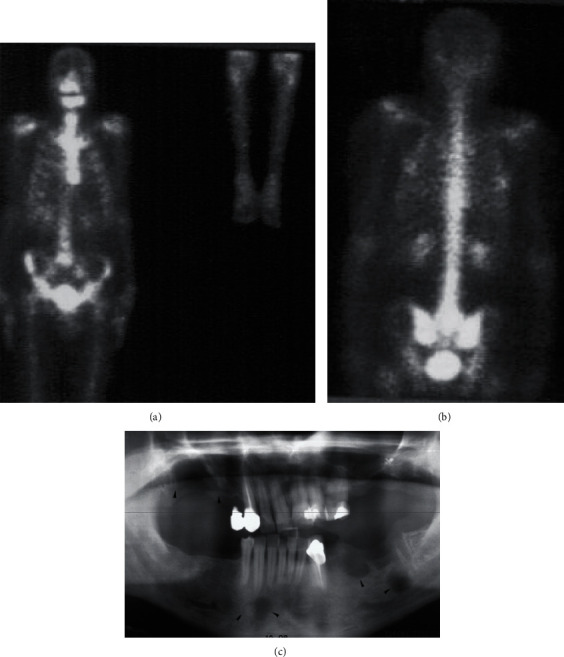
(a, b) Complete body scintigraphy with Te 99 revealing the only localization of LCH lesions in the jaws. (c) Panoramic radiograph after teeth extractions.

An entire body CT scan was accomplished which showed no evidence of lesions in other organs. Bone scintigraphy was completed and confirms that the lesions are limited in the right maxilla and on the left mandible (Figures 2(a) and 2(b)).

The treatment plan we proposed and accepted by the patient was the extraction of all the teeth in the affected region followed by fractionated stereotactic low-dose RT after two months (Figure 2(c)).

### 2.1. Radiation Therapy

A 6-megavolt linear accelerator was used with a 0.5 mm precision of its isocenter.

**Figure 3:**
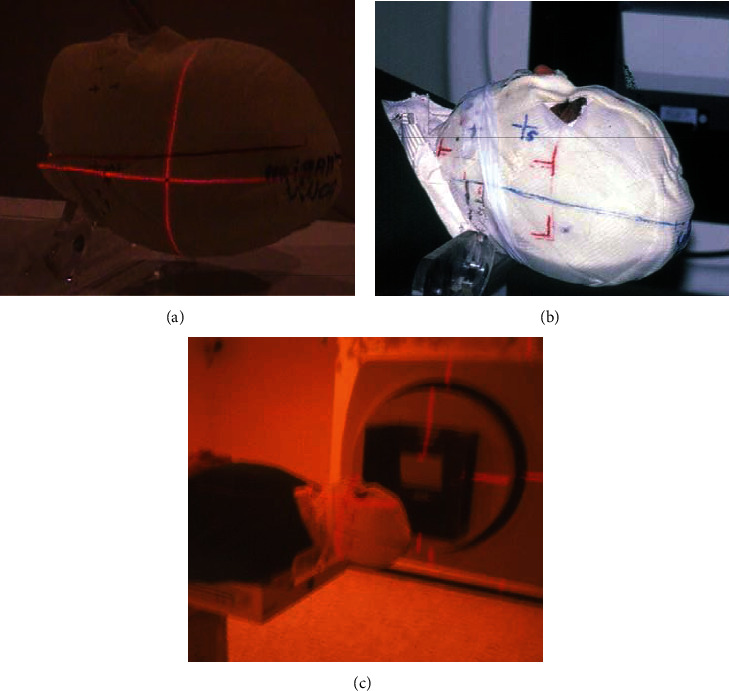
(a) Custom-made rigid mask. (b) Patient wearing the stabilisation mask. (c) 3D treatment planning is done using the CT scan images.

A custom-made rigid mask was fabricated and used for accurate immobilization of the head as described by Al Salah and El Hajj [15]. The objective of the custom-made rigid mask is to stabilize the head during the radiotherapy sessions (Figures 3(a) and 3(b)).

3D treatment planning is prepared using the CT scan images fed into the planning system (Figure 3(c)). The target volume is determinate and verified by the radiation oncologist. Dosimetry planning is organized around three isocenters for the mandible and a single isocenter for the right maxillary sinus. Planning has been done to avoid the parotid glands.

Each of the right maxilla and the left mandible received 24 Gy in 16 fractions (1.5 Gy in each session) during 32 days.

Preventive and palliative treatments included dexamethasone cream applied twice daily to the irradiated skin and mouth baths of a mixture of bicarbonate, antifungal gel, and local anesthetic application 3 times per day. Moreover, customized tooth adapted aligners were fabricated before the RT sessions for fluoride gel application for 5 minutes every day before, during, and after the end of RT treatment for 3 months. The patient presented a grade two mucositis during the treatment and was treated with corticosteroid oral rinses beside the good oral hygiene.

Xerostomia grade two has been experienced during the last two weeks of irradiation, but complete recovery has been reached one month after the treatment.

**Figure 4:**
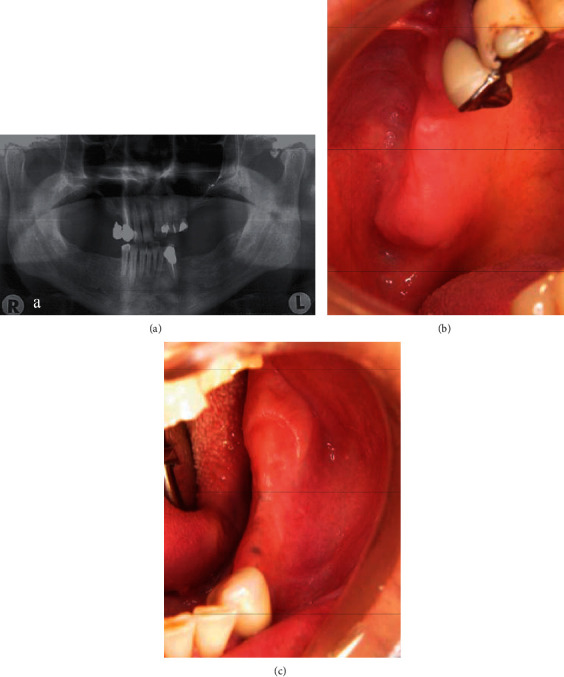
One-year follow-up after the end of RT treatment: (a) panoramic radiograph; (b) maxillary soft tissue healing; (c) mandibular soft tissue healing.

The patient was followed up in the beginning, every 6 months yearly. Complete remission was achieved clinically and radiologically after one year (Figures 4(a)–4(c) ).

**Figure 5:**
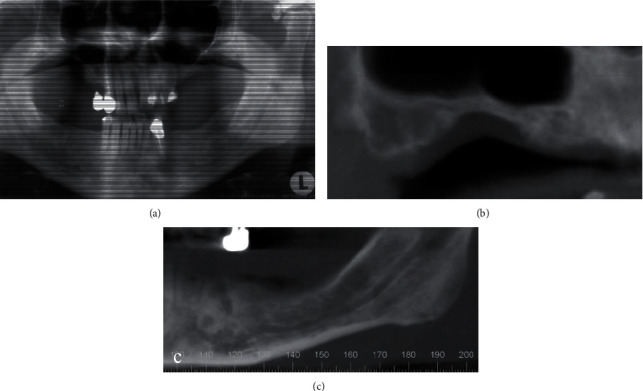
5-year follow-up radiographs: (a) panoramic radiograph; (b) sagittal CBCT image showing complete bone healing in the maxilla; (c) sagittal CBCT image showing complete bone healing in the mandible.

The panoramic X-ray and CBCT after 5 years confirm the complete bone healing process (Figures 5(a)–5(c)).

## 3. Discussion

Several treatment modalities have been reported for solitary and polyostotic bone lesions such as curettage or excision [7, 9], intralesion steroid injection [10], chemotherapy treatment with vinblastine, and prednisone [9] or cytosine arabinoside [8], and low-dose RT [11–14]. The effectivity of RT in LCH has been widely reported, but several elements should be respected such as the age of patients, the possibility of radiogenic malignancies, and the semibenign character of the disease [11–14].

It is used as a single treatment option or in combination with chemotherapy, surgery, or steroid injections [7–14].

The mechanism of action induced by ionizing radiation on target cells remains unclear [12, 13].

In adults, the recommended doses range between 10 and 45 Gy and should be supplied in fractions of 1–2 Gy by session to respect the capacity for tissue repair mechanisms [11–13].

Olschewski and Seegenschmiedt (2006), evaluated 89 LCH patients treated by RT with fractionation and 2 Gy as a median single dose and 24 Gy as median total, 91% got a local remission of the treated lesions, 77.5% had a complete remission, and only 9% developed recurrence [11].

Kriz et al. (2013) published the results of a retrospective analysis performed by the German Cooperative Group for Benign Diseases where 80 patients suffering from LCH were treated with RT during the last past 45 years and found that 77% achieved a complete remission with a median follow-up period of 54 months. They used RT with fractionation of a median total dose of 15 Gy, and the median single fraction was 2 Gy [12].

The treatment protocol proposed to our patient was very mild toxicity.

Healing of soft tissue and bone was observed over the 5-year follow-up.

## 4. Conclusion

LCH is an idiopathic benign disorder touching both genders at any age and can affect one or more organs. It is usually characterized by monostotic or polyostotic osteolytic bone lesions.

There is no consensus treatment for the management of these lesions. Fractionated stereotactic low-dose RT is deemed to be an efficient option for LCH treatment alone or combined with other treatment modalities.

## Data Availability

All the data are presented in the paper.
